# String/Cdc25 phosphatase is a suppressor of Tau-associated neurodegeneration

**DOI:** 10.1242/dmm.049693

**Published:** 2023-01-23

**Authors:** Andreia C. Oliveira, Madalena Santos, Mafalda Pinho, Carla S. Lopes

**Affiliations:** ^1^ Instituto de Investigação e Inovação em Saúde (i3S), Universidade do Porto, 4200-135 Porto, Portugal; ^2^ PhD Program in Molecular and Cell Biology, Instituto de Ciências Biomédicas Abel Salazar (ICBAS), Universidade do Porto, 4050-313 Porto, Portugal; ^3^ Instituto de Biologia Molecular e Celular (IBMC), Universidade do Porto, 4200-135 Porto, Portugal; ^4^ Department of Anatomy, Unit for Multidisciplinary Research in Biomedicine (UMIB), ICBAS, Universidade do Porto, 4050-313 Porto, Portugal; ^5^ Laboratory for Integrative and Translational Research in Population Health (ITR), 4050-600 Porto, Portugal; ^6^ Department of Pathological, Cytological and Thanatological Anatomy, ESS|P.PORTO, 4200-072 Porto, Portugal

**Keywords:** Tauopathy, Stg/Cdc25, Tau, *Drosophila*, Neurodegeneration

## Abstract

Tau pathology is defined by the intracellular accumulation of abnormally phosphorylated Tau (MAPT) and is prevalent in several neurodegenerative disorders. The identification of modulators of Tau abnormal phosphorylation and aggregation is key to understanding disease progression and developing targeted therapeutic approaches. In this study, we identified String (Stg)/Cdc25 phosphatase as a suppressor of abnormal Tau phosphorylation and associated toxicity. Using a *Drosophila* model of tauopathy, we showed that Tau dephosphorylation by Stg/Cdc25 correlates with reduced Tau oligomerization, brain vacuolization and locomotor deficits in flies. Moreover, using a disease mimetic model, we provided evidence that Stg/Cdc25 reduces Tau phosphorylation levels independently of Tau aggregation status and delays neurodegeneration progression in the fly. These findings uncover a role for Stg/Cdc25 phosphatases as regulators of Tau biology that extends beyond their well-characterized function as cell-cycle regulators during cell proliferation, and indicate Stg/Cdc25-based approaches as promising entry points to target abnormal Tau phosphorylation.

## Teaser

**Summary:** Using a *Drosophila* model of tauopathy, expressing Tau2N4R, we show that Stg/Cdc25 activity promotes Tau dephosphorylation and suppresses neurodegeneration in the fly, independently of Tau aggregation status.

## INTRODUCTION

Tauopathies are a group of neurodegenerative disorders defined by the intracellular accumulation and aggregation of Tau (MAPT), a microtubule-binding protein. These disorders include Alzheimer's disease (AD), Parkinson's disease, progressive supranuclear palsy and frontotemporal dementia, among others (reviewed in Chung et al., 2021). Although the mechanisms leading to Tau aggregation and toxicity are still not fully understood, it is recognized that changes in protein structure/solubility lead to dimer and oligomer formation, which assemble into fibrillary structures, and large insoluble aggregates (Šimić et al., 2016).

Mutations in *MAPT*, the Tau-encoding gene, and post-translational modifications (PTMs), including phosphorylation, acetylation and ubiquitination, are the most common alterations associated with Tau pathology (Limorenko and Lashuel, 2022; Tapia-Rojas et al., 2019). Recently, cryo-electron microscopy studies showed that cross-talk between PTMs underlies Tau structural diversity, affects fibril structure and correlates with distinctive tauopathies (Arakhamia et al., 2020; Fitzpatrick et al., 2017). Thus, the identification of Tau cellular partners that elicit changes in Tau structure and biology is key to understanding pathophysiological mechanisms and prompting the development of effective therapeutic approaches.

*Drosophila* tauopathy models have been successfully used to uncover Tau interactors and investigate the molecular basis of Tau pathogenesis (Limorenko and Lashuel, 2022). Pan-neuronal expression of wild-type or mutated human Tau (referred to as hTau) isoforms in flies recapitulates key pathological features of human tauopathies, including accumulation of abnormally phosphorylated forms of Tau, neuronal loss, progressive motor deficits and neurodegeneration. When expressed in the developing fly retina, hTau induces alterations in the external eye structure, characterized by the appearance of a rough eye phenotype that correlates with photoreceptor axon degeneration and loss of retinal cells (Jackson et al., 2002; Prüßing et al., 2013). This phenotype has been widely used in genetic screens and enabled the identification of cellular processes involved in Tau toxicity, which include Tau phosphorylation and proteolysis, cytoskeleton reorganization, chromatin regulation and apoptosis (Feuillette et al., 2020; Hannan et al., 2016).

Unbalanced activity of kinases and phosphatases has been associated with Tau pathology. Interestingly, Shulman and Feany (2003) reported an interaction between hTau and String (Stg), the fly CDC25 homolog, when screening for suppressors of the rough eye phenotype induced by TauV337M, a mutation associated with frontotemporal dementia and Parkinsonism linked to chromosome 17 (FTDP-17) (Shulman and Feany, 2003). Phosphatases from the CDC25 family (CDC25A/B/C) are dual-specificity phosphatases with function associated with proliferating tissues, as key regulators of cyclin-dependent kinase (CDK) activity during cell division (Rudolph, 2007). Even though neurons in the normal adult brain have exited the cell cycle, CDC25A, CDC25B and CDC25C phosphatases are expressed in this tissue, and display basal enzymatic activities (Ding et al., 2000; Vincent et al., 2001). Interestingly, increased expression and activity of CDC25A has been reported in brain tissue from AD patients (Ding et al., 2000; Vincent et al., 2001). The role of CDC25 phosphatases in neurons is still unclear, as is their link to Tau biology and neurotoxicity.

We previously showed that Stg phosphatase is expressed in photoreceptor neurons (Lopes and Casares, 2015). Here, we use a fly tauopathy model to investigate the link between Stg/Cdc25 phosphatase and Tau and explore the neuronal function of Cdc25 phosphatases. We show that Stg/Cdc25 suppresses Tau-induced phenotypes, confirming the genetic interaction previously reported. This occurs with concurrent reduction of Tau phosphorylation levels, revealing Stg/Cdc25 phosphatases as novel modulators of Tau toxicity *in vivo*.

## RESULTS

### Stg phosphatase activity suppresses Tau-induced phenotypes

To explore the genetic interaction between Stg and Tau, we used the well-established fly tauopathy model *GMR-hTau2N4R* (hereafter *GMR-hTau*), which is based on the expression of the longest hTau isoform (2N4R) under the control of the glass multimer reporter (GMR) regulatory sequences, which drive transcription in all cell types posterior to the morphogenetic furrow in the developing eye (Hay et al., 1994; Jackson et al., 2002). Accordingly, hTau is expressed in all cells of the developing retina, including photoreceptor neurons (Ellis et al., 1993). To validate Stg as a neuron-specific modifier of hTau toxicity, we used a *GMR-hTau* fly line that contains the pan-neuronal driver *elav*-Gal4 in the genetic background (*elav-Gal4; GMR-hTau*)*.* In the retina, Elav-Gal4 will drive expression of UAS sequences in photoreceptors and neurons associated with inter-ommatidia bristles, in which hTau is co-expressed.

**Fig. 1.**
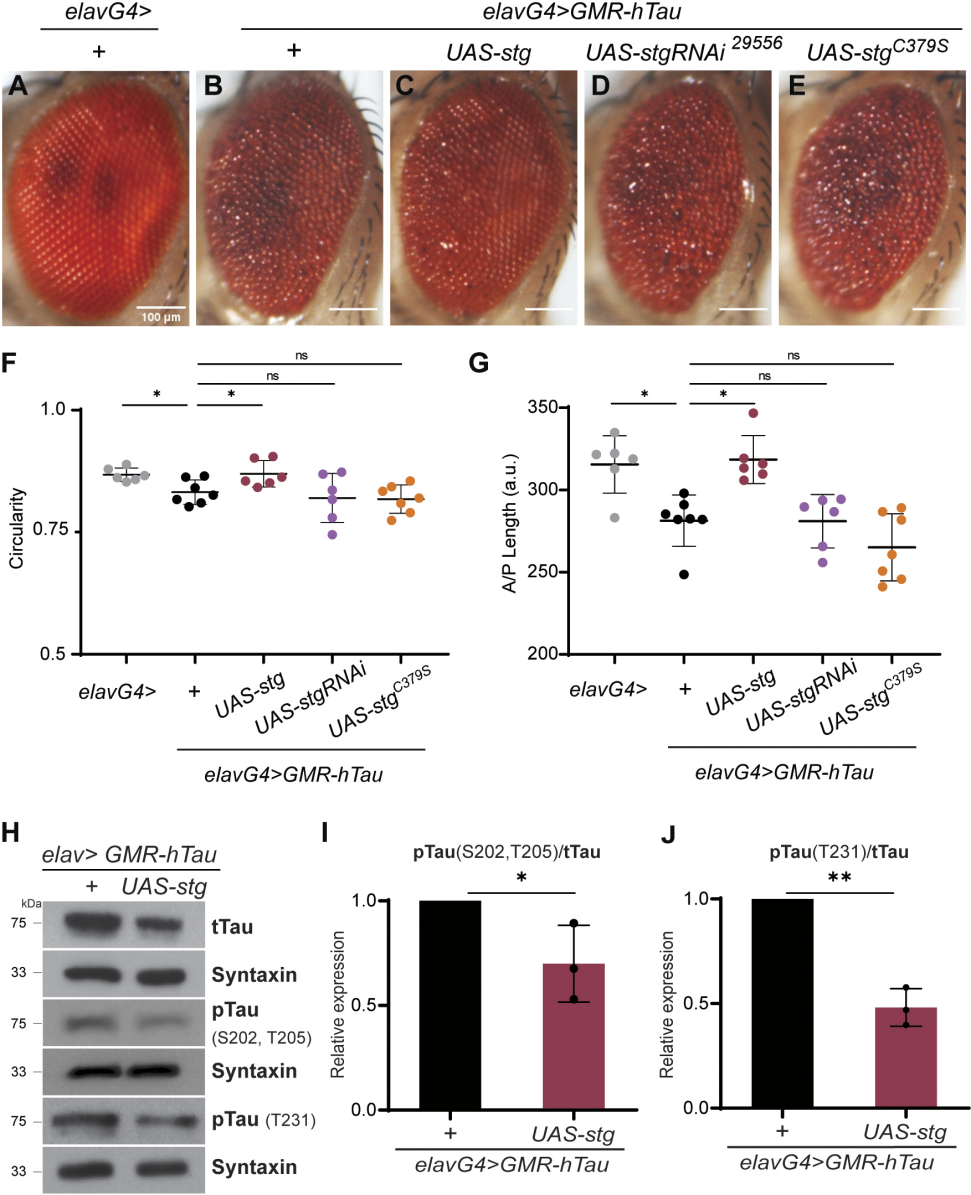
**String (Stg) phosphatase suppresses the rough eye phenotype and hyperphosphorylation associated with human Tau (hTau) expression in the fly retina.** (A-E) Representative images of adult retinas from control flies (*elavG4>+*; *n*=6) (A), flies expressing one copy of hTau (*elavG4>GMR-hTau*; *n*=7) (B), GMR-hTau flies co-expressing Stg (*elavG4>GMR-hTau; UAS-stg*; *n*=6) (C), *elavG4>GMR-hTau*; *UAS-stgRNAi* (BDSC 29556; *n*=6) flies (D) and flies expressing a phosphatase-dead *stg* allele (*elavG4>GMR-hTau*; *UAS-stg*^*C379S*^; *n*=7) (E). (F) Quantification of circularity of the retina of flies for the indicated genotypes. (G) Quantification of the anterior–posterior axis (A/P) length in the indicated genotypes. a.u., arbitrary units. In F and G, image quantification was performed using Fiji-ImageJ, and the results were analyzed using one-way ANOVA with Tukey's multiple comparison test. (H) Western blot analysis of protein extracts from fly heads of the indicated genotypes, blotted with total Tau (tTau) and Tau phosphorylated (pTau) at S202/T205 and T231. Syntaxin was used as loading control. (I,J) Quantification of the levels of pTau at S202/T205 (I) and T231 (J), normalized to tTau levels. Data refer to three replicates. Data were analyzed using unpaired Student's *t*-test with Welch's correction, two-tailed. Error bars denote s.d.; ns, non-significant; *P<0.05; **P<0.01.

We used the external morphology of the retina as a readout of the Stg–hTau interaction (Fig. 1A-G). As previously reported (Jackson et al., 2002), when compared to control flies (*elav/+*; Fig. 1A), hTau-expressing flies (*elav;GMR-hTau/+*; Fig. 1B) displayed a rough eye phenotype, with disordered ommatidia and missing or irregular mechanosensory bristles (Fig. 1B; Fig. S1). In addition, the retina was smaller than that of controls, as shown by morphometric analysis of circularity and length of the anterior–posterior (A-P) axis (Fig. 1F,G). Stg expression in hTau-expressing flies (*elav;GMR-hTau/stg*) restored retina organization and size (Fig. 1C; Fig. S1), with A-P length and circularity undistinguishable from those of control flies (Fig. 1F,G). In contrast, knocking down Stg using RNA interference (RNAi) in hTau-expressing flies (*elav;GMR-hTau/stg^RNAi^*) did not appear to affect the retina size and morphology of hTau-expressing flies (Fig. 1D,F,G; Fig. S1).

Next, we asked whether Stg phosphatase activity was required for the suppression of the *hTau* rough eye phenotype. The active site of CDC25 phosphatases is highly conserved, and modification of the conserved cysteine in the active HCX_5_R site, was shown to abolish phosphatase activity in different models (Dunphy and Kumagai, 1991; Gautier et al., 1991; Rudolph, 2007; Sohn et al., 2004). Accordingly, we generated a *stg* allele harboring a cysteine to serine (C379S) modification on the Stg conserved active site (*UAS-stg*^C379S^; Stg phosphatase dead). Our results showed that hTau; Stg^C379S^ flies had severe ommatidia disorganization, without significant changes in retina size compared to that of hTau-expressing flies (Fig. 1E-G). Altogether, our findings confirm the Stg–hTau genetic interaction and show that Stg phosphatase activity is required to suppress Tau-associated phenotypes, leading us to propose that Stg/Cdc25 phosphatases may act as regulators of Tau toxicity *in vivo*.

### Stg activity reduces Tau phosphorylation levels

We next investigated whether Stg affects Tau phosphorylation. Our analysis focused on phosphorylation of Ser202/Thr205 and Thr231 residues, which correlate with increased hTau aggregation propensity and AD clinical progression, and are used for diagnosis (Bibow et al., 2011; Hanger and Noble, 2011; Jeganathan et al., 2008; Wegmann et al., 2021). Accordingly, we compared the levels of total and site-specific phosphorylated hTau in hTau; Stg-expressing flies (Fig. 1H-J). Western blot analysis showed that hTau phosphorylation levels at Ser202/Thr205 and Thr231 residues were reduced by co-expression of *stg* in fly tissues (Fig. 1H). Quantification of the ratio between phosphorylated and total hTau indicated a reduction in phosphorylation levels in all residues under study (Fig. 1I,J). These results clearly show that Stg is able to counteract hTau phosphorylation *in vivo*.

### Endogenous Stg clusters with hTau in neurons

**Fig. 2.**
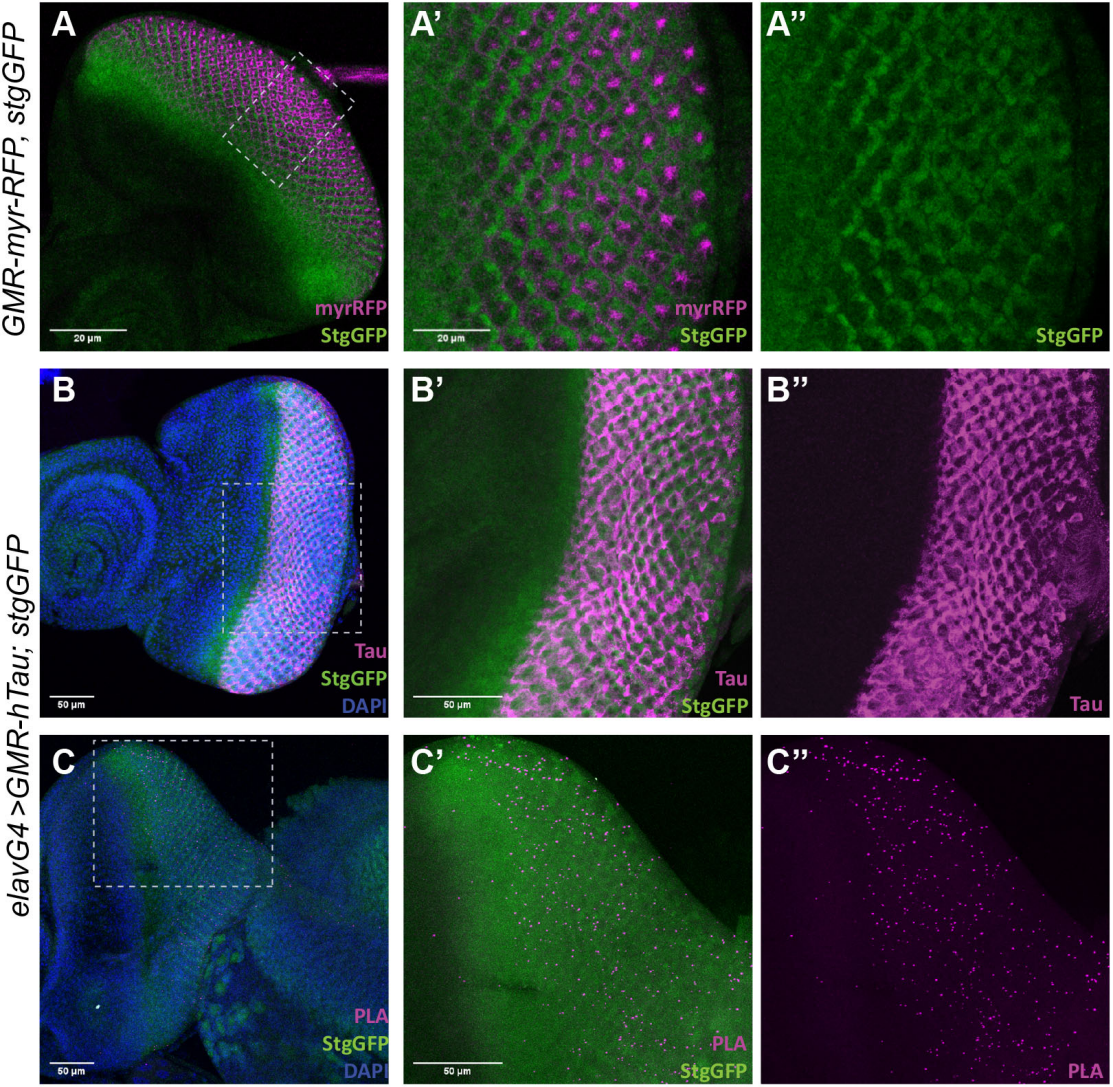
**Stg interacts physically with hTau.** (A) Representative third-instar eye imaginal disc from *GMR-myrRFP; stg-GFP* stained for GFP (green) to visualize the Stg expression pattern. *GMR-myrRFP* (magenta) labels the GMR expression domain. (A′,A″) Magnification of the region delimited by the dashed lines in A. Scale bars: 20 µm. (B) *GMR-hTau; stg-GFP* third-instar eye imaginal disc, showing the expression pattern of hTau (magenta) and Stg-GFP (Stg-GFP; green). DNA (blue) was counterstained with DAPI. (B,B″) Detail from the posterior domain of the disc shown in B. Scale bars: 50 µm. (C) L3 eye imaginal disc from *elav>GMR-hTau; stg-GFP* labeled for Stg (green) and showing proximity ligation assay (PLA) puncta (magenta), a readout of the interaction between endogenous Stg (Stg-GFP; green) and hTau. DAPI (blue) was used to counterstain DNA. (C,C″) Magnification of the posterior region of the disc, showing PLA puncta (magenta). Scale bars: 50 µm. In all images, anterior is to the left.

The reduced hTau phosphorylation levels detected upon Stg expression can be explained by direct Stg–hTau interaction. Alternatively, Stg can regulate hTau phosphorylation status indirectly, through regulation of Tau kinases and phosphatases. Because the association of protein phosphatases like Stg/Cdc25 with their substrates is transient and difficult to detect (Fahs et al., 2016; Sohn et al., 2004), we used *in situ* proximity ligation assay (PLA) to probe Stg–hTau interaction. We used a *stg* allele expressing Stg-GFP fusion under the *stg* endogenous promoter (Stg-GFP, protein trap) to evaluate Stg–Tau proximity in photoreceptors, avoiding false-positive interactions due to Stg overexpression. In third-instar eye imaginal discs, Stg-GFP was expressed at high levels in the proliferative/anterior domain and immediately posterior to the morphogenetic furrow (Fig. 2A). Immunolocalization studies in *elav;GMR-hTau/stg-GFP* larvae showed that hTau was expressed in cells posterior to the morphogenetic furrow, following the expression pattern of the GMR promoter and overlapping the Stg-GFP expression domain (Fig. 2B). PLA assays revealed the presence of *in situ* PLA signals in the domain co-expressing Stg and hTau (Fig. 2C), which indicated that Stg-GFP and hTau were found in proximity in these cells. In contrast, no PLA signals were observed in eye-imaginal discs from *GMR-hTau; TM3,Ser* larvae, confirming the specificity of *in situ* PLA detection (Fig. S2).

### Stg suppresses Tau-driven neurodegeneration

Well-established neurodegeneration phenotypes like cell death (Jackson et al., 2002; Wittmann et al., 2001), brain vacuolization (Khurana et al., 2006; Mershin et al., 2004; Wittmann et al., 2001) and locomotor impairment (Sealey et al., 2017; Ubhi et al., 2007) are strongly associated with pan-neuronal expression of hTau and phosphorylation status (Nishimura et al., 2004). Therefore, we asked whether Stg expression would modify neurodegeneration signatures in hTau-expressing flies.

**Fig. 3.**
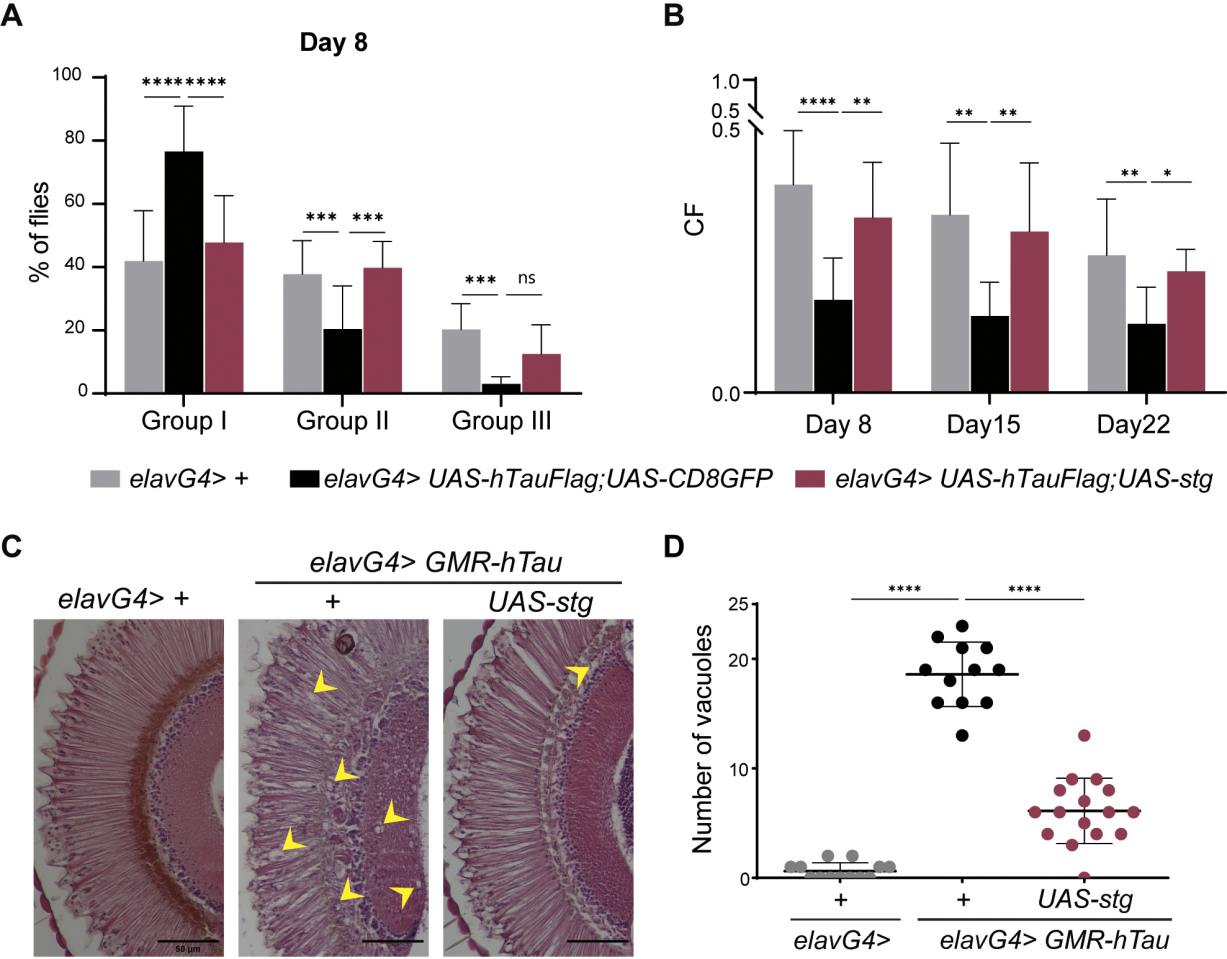
**Stg rescues hTau-associated neurodegeneration phenotypes.** (A) Quantification of the percentage of flies retained in group I (severe climbing defects), II (moderate climbing defects) and III (reduced climbing defects) at day 8 for *elavG4* (*n*=346), *elavG4>UAS-hTau; UAS-CD8GFP* (*n*=316) and *elavG4> UAS-hTau; UAS-stg* (*n*=226). (B) Graphic representation of the climbing index [partition coefficient (CF); probability of flies to climb] for 8, 15 and 22 days of the indicated genotypes. Statistical significance was calculated using two-way ANOVA, multiple comparisons. (C) Representative cross-sections of adult retinas from control (*elavG4/+*), hTau (*elavG4>GMR-hTau/+*) and hTau; Stg (*elavG4>GMR-hTau; UAS-stg*)-expressing flies, stained with Hematoxylin and Eosin. Yellow arrowheads indicate vacuoles. Scale bars: 50 µm. (D) Quantification of the number of vacuoles in the lamina with a diameter ≥3.5 µm for *elavG4* (*n*=13), *elavG4>GMR-hTau* (*n*=12) and *elav;GMR-hTau; UAS-stg* (*n*=16). Statistical significance was calculated using one-way ANOVA with Tukey's multiple comparison test. Error bars denote s.d.; ns, not significant; *P<0.05; **P<0.01; ***P<0.001; ****P<0.0001.

To assay locomotor function, we performed a climbing assay in 8-, 15- and 22-day-old flies using a countercurrent apparatus (Fig. S4). In this assay, we evaluated a fly’s ability to climb up a tube, in a set period of time, in five consecutive attempts (Inagaki et al., 2010). Flies that successfully climbed up the tube were transferred to a new tube and challenged again. Thus, flies reaching the last tubes, 5 and 6 (group III), had increased climbing ability compared to those retained in tubes 1 and 2 (group I), despite being given the same five climbing attempts. We observed that the climbing performance of hTau-expressing flies was significantly impaired (Fig. 3A,B), in agreement with previous observations (Sealey et al., 2017). By day 8, only 3% of hTau-expressing flies had reached tubes 5 and 6 (group III), while 77% were retained in group I (tubes 1 and 2) (Fig. 3A). In contrast, at the same time point, more than 10% of hTau; Stg-expressing flies had completed the assay (group III) (Fig. 3A). Their behavior was similar to that observed in control flies. Moreover, the climbing probability for each genotype, given by the partition coefficient (CF), showed that, over time, hTau; Stg flies performed better than hTau flies (Fig. 3B). The improved locomotor function associated with Stg expression was sustained, and, by day 22, hTau; Stg-expressing flies remained indistinguishable from controls (Fig. 3B; Fig. S4). Although the climbing performance decreased with time in all genotypes, most likely due to age-related deterioration, Stg co-expression increased CF to values similar to those of control at all time points analyzed (Fig. 3B).

Next, we evaluated the internal morphology of the retina using standard Hematoxylin and Eosin staining (Fig. 3C). Analysis of adult head sections from GMR-hTau flies revealed severe disruption of the internal structure of the visual system, with strong vacuolization in both retina and lamina regions (Fig. 3C). Importantly, expression of Stg in photoreceptors significantly improved the internal structure of the retina of GMR-hTau flies (Fig. 3C). In addition, quantification of the number of vacuoles between GMR-hTau and GMR-hTau; Stg-expressing flies revealed a strong reduction in vacuole number in GMR-hTau; Stg 10-day-old flies (Fig. 3D). These observations indicate that Stg expression suppresses Tau-associated neurodegeneration phenotypes and suggest that the dephosphorylation mediated by Stg reduces hTau cellular toxicity.

### Oligomerization and Tau spreading is impaired by Stg activity

**Fig. 4.**
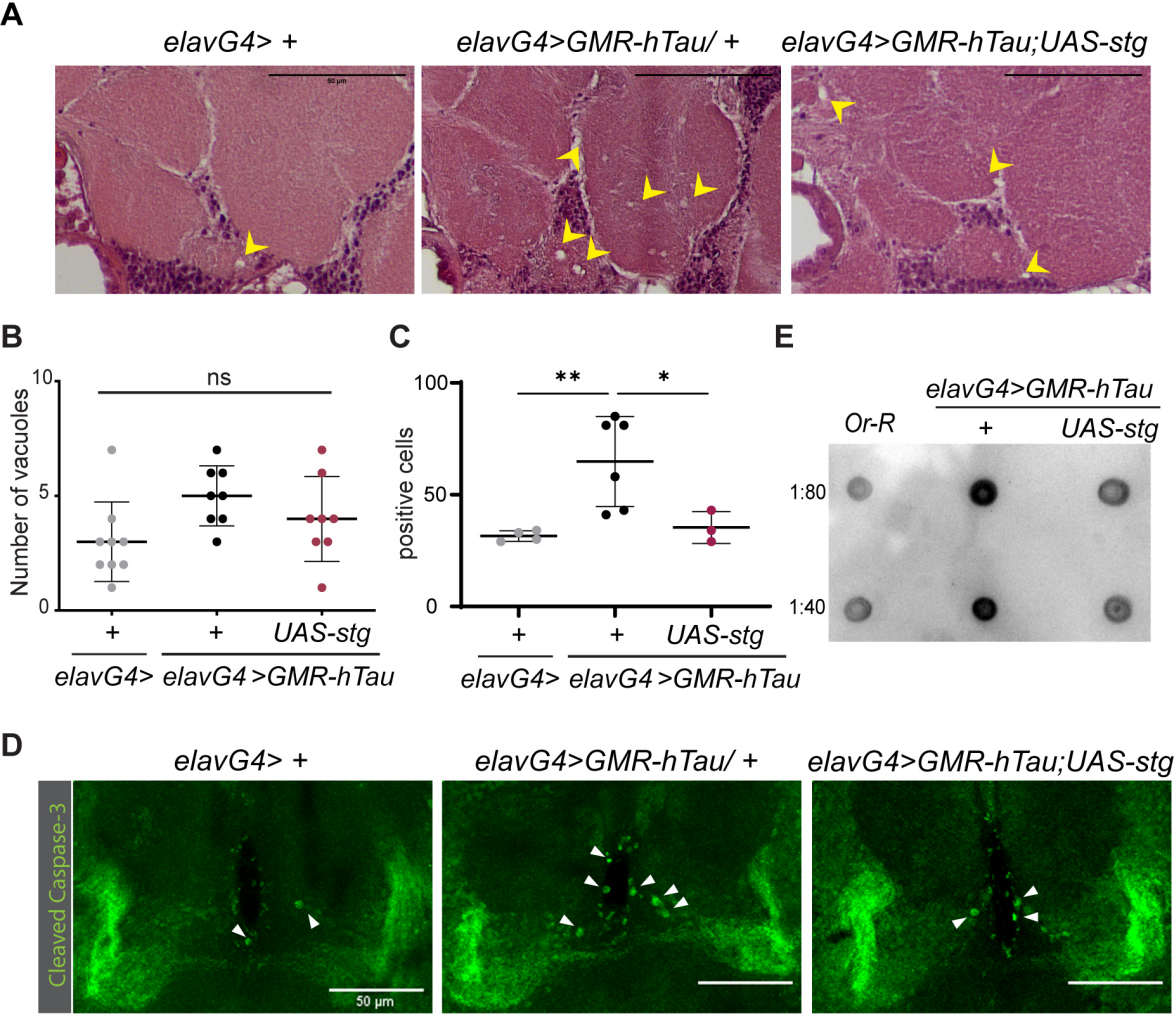
**Stg reduces the levels of hTau toxic species.** (A) Representative cross-sections of adult heads from control (*elavG4>+*), and hTau (*elavG4> GMR-hTau;+*)- and hTau; Stg (*elavG4;>GMR-hTau; UAS-stg*)-expressing flies, stained with Hematoxylin and Eosin. Vacuoles are indicated by yellow arrowheads. Scale bars: 50 µm. (B) Quantification of the number of vacuoles in the central brain region of *elavG4* (*n*=9), hTau (*n*=8) and hTau; Stg (*n*=8) 10-day-old flies. (C) Quantification of Caspase-3-immunoreactive cells in brain of *elavG4>+* (*n*=4), *elavG4>GMR-hTau;+* (*n*=6) and *elavG4;>GMR-hTau; UAS-stg* (*n*=3) 10-day-old flies. (D) Representative images of adult fly brains stained for Caspase-3. Caspase-3-positive cells are indicated by white arrowheads. Scale bars: 50 µm. (E) Dot blot analysis of oligomeric species in soluble fraction of extracts from fly heads of the indicated genotypes. Two dilutions of extract were analyzed (*n*=3). Statistical significance was calculated using one-way ANOVA with Tukey's multiple comparison test. Error bars denote s.d.; ns, non-significant; *P<0.05; **P<0.01.

During the analysis of adult head sections, we consistently detected the presence of vacuoles in the central brain region of *GMR-hTau* flies (Fig. 4A). This was unexpected because hTau expression is limited to cells within the posterior domain of the eye imaginal disc (Fig. 2B) and is not expressed in brain neurons. The occurrence of vacuolization in the central brain area is suggestive of spreading of toxic forms of hTau from the retina to neighboring regions in the fly head. To address this hypothesis, we quantified the number of vacuoles in the central brain region and apoptotic cells using the cleaved Caspase-3 marker, in all genotypes under study (Fig. 4B). Our analysis showed that Stg expression reduced vacuolization tendency in hTau-expressing flies (Fig. 4A,B). Quantification of cell death, after cleaved Caspase-3 labeling, revealed increased apoptosis in *GMR-hTau* relative to control flies (Fig. 4C,D). Importantly, the number of apoptotic cells was significantly reduced upon Stg expression (Fig. 4C,D). These findings support the hypothesis that hTau toxicity extends beyond the retina and imply spreading of hTau toxic forms in the *GMR-hTau* tauopathy model. Accordingly, we evaluated the presence of oligomers in extracts from hTau-expressing flies, as these are strongly implicated in hTau toxicity and the most amenable for spreading (Mudher et al., 2017). Dot blot analysis with A11, an oligomer-specific antibody (Abskharon et al., 2020), revealed strong reactivity with GMR-hTau extracts (Fig. 4E)*.* This contrasts with lower reactivity, in GMR-hTau; Stg extracts, for equivalent levels of total protein. Altogether, these findings show that Stg effects on hTau phosphorylation correlate with reduced oligomerization potential and neurodegeneration.

### Stg suppresses neurotoxicity after Tau aggregation is established

**Fig. 5.**
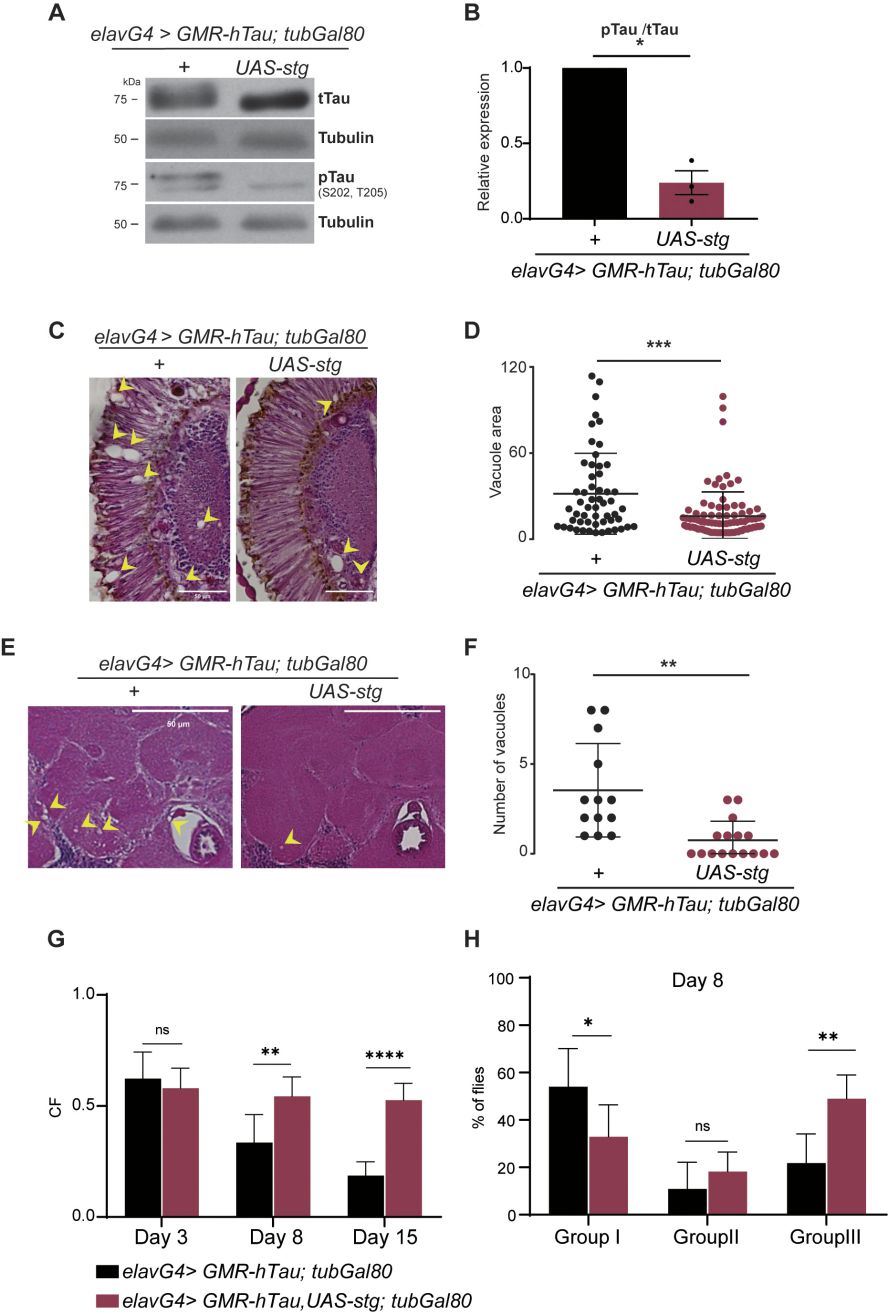
**Stg promotes dephosphorylation of hTau toxic species delaying neurodegeneration progression.** (A) Representative immunoblots of extracts from 10-day-old adult flies expressing hTau (*elavG4>GMR-hTau; tubGal80^ts^*) or hTau; Stg (*elavG4; GMR-hTau,UAS-stg; tubGal80^ts^*) in adult stages, probed with tTau and pTau. (B) Quantification of the ratio between pTau at Ser202/Thr205 and tTau levels. Tubulin was used as loading control. Data represent three replicates. The results were analyzed using unpaired Student's *t*-test with Welch's correction, two-tailed. (C,E) Representative images from retina (C) and central brain (E) of 10-day-old flies, of the indicated genotypes, stained with Hematoxylin and Eosin. Vacuoles are indicated by yellow arrowheads. Scale bars: 50 µm. (D) Quantification of vacuole size (diameter ≥3.5 µm) in the retina and lamina regions. (F) Quantification of the number of vacuoles in the central brain region for *elavG4>GMR-hTau; tubGal80^ts^* (*n*=13) and *elavG4; GMR-hTau,UAS-stg; tubGal80^ts^* (*n*=16). For D and F, the results were analyzed using unpaired Student's *t*-test with Welch's correction, two-tailed. (G) Graphic representation of the climbing index (CF) at 3, 15 and 22 days of the indicated genotypes. (H) Quantification of the percentage of flies retained in group I (severe climbing defects), II (moderate climbing defects) and III (reduced climbing defects) at day 8 for *elavG4>GMR-hTau; tubGal80^ts^* (*n*=246) and *elavG4; GMR-hTau,UAS-stg; tubGal80^ts^* (*n*=191). In G and H, statistical significance was calculated using two-way ANOVA, multiple comparisons. Error bars denote s.d.; ns, not significant; *P<0.05; **P<0.01; ***P<0.001 ***P<0.0001.

The results presented thus far highlight Stg phosphatase as a regulator of hTau phosphorylation and toxicity with effective physiological improvement in neurodegeneration progression. We next asked whether the upregulation of Stg activity would be an advantage in a disease context, as knowing this will allow the community to put forward therapeutic approaches built upon Tau regulation by Stg/Cdc25 phosphatases. To test this possibility, we assayed whether overexpressing Stg after Tau hyperphosphorylation and aggregation are established would reduce Tau-associated toxicity. We used the Gal80^ts^ system to block the expression of UAS-Stg until *elav>GMR-hTau* individuals reached adult stage*.* Analysis of protein levels in head extracts from 10-day-old flies showed a significant reduction in hTau phosphorylated at Ser 202/Thr205 after Stg upregulation (Fig. 5A,B). This clearly showed that Stg phosphatase can dephosphorylate hyperphosphorylated oligomeric forms of hTau *in vivo*.

We next asked whether the lower levels of phosphorylated hTau correlate with slower disease progression, using vacuolization of the retina and brain as a readout (Fig. 5C-F). As the retina is completely formed when flies eclose, Stg overexpression failed to restore the internal structure of the retina in 10-day-old flies (Fig. 5C). However, it was sufficient to promote a reduction in the vacuole area (Fig. 5D). Analysis of the brain region indicated that Stg expression significantly reduced the number of vacuoles detected (from 3.5 to 0.75 vacuoles, on average) (Fig. 5E,F). We next assayed the climbing ability of hTau individuals at days 3, 8 and 15 after Stg upregulation (Fig. 5G,H). Analysis of the CF showed that hTau and hTau; Stg individuals were identical at day 3 (Fig. 5G). However, following Stg continuous expression, by days 8 and 15, hTau; Stg expressing flies performed significantly better than hTau flies (Fig. 5G), suggesting that increased Stg levels and activity prevent decay of locomotor function in hTau flies. Indeed, analysis by performance groups showed that, by day 8, 48% of hTau; Stg-expressing flies were able to reach tubes 5 and 6 (group III), versus 22% of hTau (*hTau; tubGal80*) individuals (Fig. 5H). Overall, our findings show that Stg is able to promote dephosphorylation of aggregated and abnormally phosphorylated hTau, and this has a physiological impact, as indicated by improved locomotor function of hTau flies.

## DISCUSSION

Here, we show that Stg, a conserved member of the Cdc25 phosphatase family, is a suppressor of Tau- associated toxicity and neurodegeneration. Using a fly model of Tau2N4R tauopathy, we demonstrate that neuron-specific upregulation of Stg activity reduces hTau phosphorylation levels and suppresses hTau-associated phenotypes, including rough eye, locomotor deficits and brain neurodegeneration. Our work provides evidence for an unambiguous Stg–Tau genetic interaction, expanding the former identification of Stg as a genetic modifier of TauV337M phenotype, a mutation associated with familial frontotemporal dementia (Shulman and Feany, 2003). Importantly, these findings highlight a novel role for Stg/Cdc25 phosphatases as Tau regulators, with impact on disease outcome.

CDC25 phosphatases (CDC25A/B/C) play essential roles during normal cell division as regulators of CDK activity, and their increased activity is associated with cell proliferation and tumor progression (Boutros et al., 2006, 2007). Interestingly, increased expression of CDC25A and CDC25B was detected in samples from AD brains, and both were shown to accumulate in the cytoplasm of degenerating neurons (Ding et al., 2000; Vincent et al., 2001). CDC25 upregulation correlated with increased phosphatase activity towards CDK1, and the occurrence of mitotic figures in degenerating neurons (Ding et al., 2000; Vincent et al., 2001). Likewise, increased activity of CDC25A was linked to hypoxia-induced neuronal death, and correlated with increased PRB phosphorylation (Iyirhiar et al., 2017). The upregulation of cell cycle genes, including *Cdc25*, is regarded as a neuronal response to insults, as DNA damage, that promotes aberrant cell-cycle re-entry, and ultimately leads to neuronal death (Chang et al., 2012; Marlier et al., 2020). However, our findings suggest that increased expression of Cdc25 in neurons can be neuroprotective. In agreement, Cdc25 overexpression promotes regeneration in *Drosophila* sensory neurons after physical injury, whereas Cdc25 knockdown impedes regeneration (Li et al., 2021). In fact, Cdc25 participates in the evolutionarily conserved Piezo–Atr–Chek1–Cdc25 inhibitory regeneration pathway. We propose that Cdc25 may play a neuroprotective role during early steps of Tau-induced neurodegeneration, in response to compromised axonal dynamics and homeostasis, whereas at later stages of disease the co-occurrence of DNA damage elicits cell-cycle re-entry culminating in neuronal death.

Consistent with a potential homeostatic function for Cdc25 phosphatases in neurons, CDC25A and CDC25B expression has been reported in human brain samples (Ding et al., 2000; Vincent et al., 2001), mouse (Iyirhiar et al., 2017) and rat (Chang et al., 2012). The Stg/Cdc25–Tau interaction, herein described, supports function as cytoskeleton regulators. Importantly, the functional conservation between Stg and mammalian Cdc25 phosphatases predicts that Stg–Tau interaction is likely to be conserved throughout evolution. Accordingly, we propose that Stg/Cdc25 is a Tau phosphatase with strong impact on Tau biology and pathology, and its activity deserves further studies. Thus, Stg/Cdc25 adds to the list of key Tau regulators like Shaggy/GSK3-B (Jackson et al., 2002), Par-1/MARK (Ambegaokar and Jackson, 2011; Nishimura et al., 2004), MAPK and PP2A phosphatases (Shulman and Feany, 2003) identified in flies (Gistelinck et al., 2012; Limorenko and Lashuel, 2022).

Our findings show that the Stg/Cdc25 phosphatase counteracts hTau-associated toxicity. We detected a correlation between reduction in Tau phosphorylation levels mediated by Stg and suppression of neurodegeneration phenotypes. In addition, Tau dephosphorylation correlated with reduced oligomerization in fly brains. These findings suggest that Stg/Cdc25 promotes dephosphorylation of residues that directly affect Tau structure and biology. Accordingly, Hanger and Noble (2011) detected reduced phosphorylation levels at hTau residues abnormally phosphorylated in AD brains. The close proximity detected among endogenous Stg and hTau alludes to a direct interaction between the two proteins. Therefore, we put forward the hypothesis that Tau dephosphorylation is likely to be the outcome of direct Stg/Cdc25 activity. However, indirect regulation of Tau phosphorylation status, through modulation of Tau kinase(s) activity, cannot be ruled out.

Importantly, our observations unveil the potential of therapeutic approaches based on Cdc25–Tau interaction. In contrast to studies based on co-expression approaches, during fly development or restricted to adult stages (Cowan et al., 2010; Fernandez-Funez et al., 2015; Hannan et al., 2016), here we use a paradigm of disease and induce Stg/Cdc25 expression after high levels of phosphorylated Tau build-up in neurons. This approach enabled us to show that Stg/Cdc25 is able to promote dephosphorylation of highly phosphorylated toxic forms of hTau. Moreover, we observed a reduction in vacuolization levels in the brain of 10-day-old flies, in support of lagged neurodegeneration. This occurs albeit the maintenance of high levels of hTau protein. These findings show that it is the presence of highly phosphorylated soluble hTau that is detrimental to neurons, in agreement with other studies in flies and mice (Cowan et al., 2015; Feuillette et al., 2010).

Our results are in line with the hypothesis that abnormal hTau phosphorylation increases hTau toxicity and spreading potential. In flies, only the lamina and medulla neuropils receive direct input from photoreceptor neurons (Fischbach and Dittrich, 1989; Sanes and Zipursky, 2010); however, we detected increased cell death and vacuolization in the central brain of *GMR-hTau* flies. This was unexpected because in the GMR-hTau model, Tau expression is restricted to the presynaptic terminal of photoreceptors. Thus, brain vacuolization can only be explained by spreading of Tau toxic forms, most likely oligomeric Tau, to other brain regions. This appears to be the case, as we were able to detect deposits of phosphorylated hTau in the brain of both hTau and hTau; Stg flies (Fig. S3). Importantly, Tau dephosphorylation promoted by Stg correlated with reduced apoptosis and vacuolization levels in the brain of hTau flies. Similar findings were reported in mice by Hu et al. (2016), who proposed that dephosphorylation of AD-hyperphosphorylated Tau shows reduced propagation in the brain. Although the cellular mechanism(s) underlying trans-cellular Tau propagation remains to be fully identified, *Drosophila* models such as the one used in this study can provide important contributions on the mechanisms (synaptic vesicles, endocytosis, diffusion of free Tau or oligomeric forms) and molecular players involved. We expect that future studies following up on our findings will establish Cdc25 phosphatases as important regulators of Tau biology and conceivable venues to explore the development of effective therapeutic approaches.

## MATERIALS AND METHODS

### Fly strains and genetics

Flies were maintained in standard media, at 25°C under a 12:12[Supplementary-material sup1]h light–dark cycle, unless indicated. The strains *elav^C155^*GAL4 (BDSC 458), *UAS-stg* (BDSC 4777), *UAS-stg.HA* (BDSC 56562), *elav^C155^*GAL4; GMR-hTau (BDSC 51360), tubGAL80^ts^ (BDSC 7017), *UAS-stgRNAi* (BDSC 34831), *stg-GFP* (BDSC 50879), GMR::myrRFP (BDSC 7121), UAS-mCD8GFP (BDSC 5130), UAS-*stg*RNAi lines, *stg^JF03235^* (BDSC 29556), *stg^HMS00146^* (BDSC 34831), *stg^GL00513^* (BDSC 36094), UAS-*mcherry*RNAi (BDSC 35785), UAS-*mcherry* (BDSC 35787) and Oregon R were obtained from the Bloomington *Drosophila* Stock Center (NIH P40OP018537). *UAS-hTau-Flag* was previously described (Kosmidis et al., 2010). Standard genetic techniques and fly lines carrying balancers on the second and third chromosomes were used to generate the different genetic backgrounds. Control flies in all experiments were as closely related to the experimental flies as possible. *elav^C155^*Gal4 flies were crossed to *Oregon R* to generate heterozygous control alleles. Flies of genotypes containing the Gal4/UAS/Gal80^ts^ constructs were grown at 18°C and transferred to 29°C as 1- to 3-day-old adults, to allow expression of the Gal4, and aged until use in histological or protein analysis. A list of the genotypes analyzed is provided in Table S1. The phenotype of adult retinas was imaged in a stereomicroscope Stemi 2000 Zeiss equipped with a Nikon Digital SMZ 1500 camera, at 50× magnification.

### Generation of transgenic flies

The *UAS-stg^C379S^* transgenic flies were generated by replacing the conserved cysteine within the catalytic domain to serine using an NZYMutagenesis kit (NZYTech) according to the manufacturer's protocol. The following oligonucleotides were used to amplify the *stg* coding sequence from LD47579 plasmid, stgPPAseFW (5′-CAACATCATTATCTTCCACGCCGAATTCTCCTCGGAGCGT-3′) and stgPPAseRW (5′-ACGCTCCGAGGAGAATTCGGCGTGGAAGATAATGATGTTG-3′), followed by DpnI digestion of parental DNA (LD47579). After transformation of DH5α competent cells, plasmid DNA was amplified, and at least three clones were selected for sequencing analysis. EcoR1-XhoI digestion was used to clone the *stgC379S* sequence into *pUAST-attB*. Transgenic flies were obtained after site-specific integration of *UAST-stg^C379S^* on the third chromosome (3R-86F) (Bischof et al., 2007).

### Protein extraction and analysis

For protein analysis, adult flies of the appropriate genotypes were aged for 8-15 days, at the appropriate temperature, flash frozen in liquid nitrogen and stored at −80°C. Flies were decapitated by vigorously vortexing for 15 s, and heads were homogenized in ice-cold RIPA buffer (50 mM Tris-HCl, 150 mM NaCl, 1% Triton X-100), supplemented with protease and phosphatase inhibitors (Roche), in a ratio of 20 µl buffer per ten fly heads. Extracts were incubated for 1 h at 4°C with rotation and centrifuged at 2000 ***g*** for 20 min, to separate soluble from insoluble fractions. Total protein content was quantified by the Lowry Method (DC™ Protein Assay, Bio-Rad). Fifteen micrograms of soluble extract were loaded in 10% SDS-polyacrylamide gels and transferred onto nitrocellulose membranes for western blot analysis. Extracts were loaded in replicated gels and probed in parallel with total Tau and phosphorylation-specific Tau antibodies. Membranes were blocked for 1 h with Tris-buffered saline with 0.1% Tween 20 (TBST) containing either 5% low-fat milk or 5% bovine serum albumin (BSA; Sigma-Aldrich). Primary antibodies were diluted in TBST with 3% blocking agent: phospho-Tau (Ser202, Thr205) (AT8) (1:2000; Thermo Fisher Scientific), phospho-Tau (Thr231) (AT180) (1:2000; Thermo Fisher Scientific), total Tau (5A6) [1:6000; Developmental Studies Hybridoma Bank (DSHB)], anti-Syntaxin (1:500; DSHB) and anti-Tubulin (1:10,000; DSHB). Goat anti-rabbit and goat anti-mouse horseradish peroxidase-conjugated secondary antibodies (Amersham) were diluted at 1:10,000 and 1:15,000, respectively, in TBST containing 3% low-fat milk. Signal was detected using Clarity Western ECL Substrate (Bio-Rad) according to the manufacturer's instructions. A GS-800-calibrated densitometer with Quantity One 1-D Analysis Software 4.6 (Bio-Rad) was used for quantitative analysis of protein levels. Western blots were repeated at least three times with biological replicates, and representative blots are shown (Fig. S5).

### Dot blot analysis

For dot blot analysis, ten fly heads were homogenized in RIPA buffer supplemented with protease and phosphatase inhibitors. After a 5 min centrifugation at 10,000 ***g***, samples were diluted in 1% glycerol/PBS. Then, 3 µl of protein was dotted in nitrocellulose membrane. After blocking in 5% milk/TBST, the membrane was incubated with anti-amyloid oligomer A11 antibody (1:500; AB9234, Merck) overnight at 4°C and handled for protein detection following the protocol described above. Images were acquired in ChemiDoc XRS (Bio-Rad).

### Histology analysis

Female flies were anesthetized on ice, immobilized in a histology collar and fixed with Carnoy's solution (60% ethanol, 30% chloroform and 10% glacial acetic acid), overnight at 4°C. Tissue was dehydrated in an ethanol series prior to paraffin embedding and microtome sectioning as described previously (Sunderhaus and Kretzschmar, 2016). Serial sections (5 μm) from the entire head were stained with Hematoxylin and Eosin and examined by bright-field microscopy. Images were captured with an Olympus DP72 microscope. Total vacuole number and vacuole area were quantified in the central brain, retina and lamina regions. For the immunocytochemical analyses, head sections were mounted on adhesive microscope slides (StarFrost, Knittel Glass) and immunostained using the modified avidin–biotin–peroxidase complex (ABC) method. Antigen retrieval was performed by microwave treatment in 0.01 M citrate buffer at pH 6.0 for 10 min. Endogenous peroxidase was blocked by treatment with 3% hydrogen peroxide in methanol for 20 min. After washing with PBS, 0.05% Tween 20, the sections were incubated for 20 min in a humid chamber with normal rabbit serum (X092, Dako) diluted 1:5 in PBS, 0.05% Tween 20 with 10% BSA. The sections were then incubated overnight at 4°C with phospho-Tau (AT8; 1:500) antibody diluted in PBS, 0.05% Tween 20, 5% BSA. Tissue was incubated for 30 min with a biotin-labeled rabbit anti-mouse secondary antibody (EO35301-2, Dako) diluted 1:200 in PBS, 0.05% Tween 20, 5% BSA followed by incubation in an ABC (1:100 in 5% BSA; Vector Laboratories) for an additional 30 min. The slides were incubated with 3,3′-diaminobenzidine tetrahydrochloride (DAB; Dako). Tissue sections were counterstained with Mayer's Hematoxylin (HX390929, Merck), and dehydrated slides were mounted in Entellan.

### Immunofluorescence analysis

Third-instar larval tissues were dissected and fixed with 3.7% formaldehyde in PBS for 20 min, followed by washes in PBS with 0.1% Triton X-100 (0.1% PBT). Primary antibodies used were anti-GFP (1:1000; A11122, Invitrogen) and anti-Tau (1:5000; 5A6, DSHB). Secondary antibodies conjugated with Alexa Fluor dyes (Thermo Fisher Scientific) were diluted 1:1000 in 0.1% PBT. Samples were mounted in 50% glycerol/PBS. Brains from 10-day-old flies were dissected in cold PBS and fixed in 3.7% formaldehyde in PBS for 30 min. Samples were washed three times in PBS with 0.5% Triton X-100 (0.5% PBT; Sigma-Aldrich) and incubated in blocking buffer (0.5% PBT+0.5% BSA+0.5% FBS) for 90 min at room temperature. Incubation with primary antibodies anti-cleaved Caspase-3 (Asp 175) (1:50; Cell Signaling Technology) and anti-ELAV (1:400; DSHB) was performed in blocking buffer overnight at 4°C. Secondary antibodies conjugated with Alexa Fluor dyes (Thermo Fisher Scientific) were diluted 1:1000 in 0.5% PBT and incubated for 3 h at room temperature. Tissues were mounted in Fluoromount-G™ Mounting Medium (Thermo Fisher Scientific). Samples were imaged on a Leica SP5 confocal microscope, and images were processed using ImageJ [National Institutes of Health (NIH)].

### PLA

The Duolink In Situ Red system (92101, Merck) was used to detect Stg–Tau interaction *in vivo*, following the manufacturer's protocol. Briefly, third-instar larvae eye imaginal discs were dissected and fixed as described previously, and incubated with Duolink Blocking Solution for 1 h at 37°C. Incubation with the primary antibodies anti-GFP (1:1000; A11122, Invitrogen) and anti-hTau (1:5000; 5A6, DSHB) was performed overnight at 4°C. Samples were washed twice with Duolink Wash Buffer A, and incubated with MINUS (anti-mouse) and PLUS (anti-rabbit) Duolink PLA Probes for 1 h at 37°C. Samples were washed twice with Duolink Wash Buffer A prior to the ligation (1 h at 37°C) and amplification (90 min at 37°C) steps . Eye imaginal discs were mounted in Duolink In Situ Mounting Medium with 4′,6-diamidino-2-phenylindole (DAPI; Merck). Images were acquired using a Leica SP5 confocal microscope.

### Climbing assays

Climbing assays were performed in a countercurrent apparatus equipped with six chambers, as described in Inagaki et al. (2010). Newly hatched flies of the appropriate genotypes were allowed to mate for 2 days, and then separated by sex in groups of 10-15 flies and aged at 25°C. At each time point, flies were placed into the first chamber, tapped vigorously to the bottom and given 15 s to climb upwards (∼10 cm), reaching the upper tube. The flies that successfully reached the upper tube were shifted to a new chamber, and both sets of flies were given another opportunity to climb upwards, in 15 s. This procedure was repeated a total of five times, and the number of flies in each chamber was counted. Flies were classified into three performance groups according to the locomotor ability displayed: flies that remained in tubes 1 and 2 were considered to have severe locomotor impairment (group I); those remaining in tubes 3 and 4 were considered to have moderate locomotor impairment (group II); and those that reached the last tubes, tubes 5 and 6, were considered to perform well in the climbing assay (group III). Climbing assays were performed at days 8, 15 and 22, and the CF was calculated, according to Inagaki et al. (2010). CF represents the probability of flies to climb. At least 100 flies were used per genotype.

### Scanning electron microscopy (SEM)

For SEM analysis, 1-day old flies were dehydrated through incubation in ethanol series (25%, 50%, 75%, 100%), incubated with hexamethildizilasane (HMDS; Sigma-Aldrich), air dried, mounted in SEM stubs and coated with Au/Pd thin film, by sputtering using the SPI Module Sputter Coater equipment. Imaging was performed using a High-Resolution (Schottky) Environmental Scanning Electron Microscope (Hitachi) with X-ray microanalysis and electron backscattered diffraction analysis (Quanta 400 FEG ESEM/EDAX Genesis X4M). Images were acquired at 400× and 1000× magnification.

### Statistical analysis

For western blot analysis, significance was determined using unpaired Student's *t*-test with Welch's correction, two-tailed. For the climbing assay, significance was measured using two-way ANOVA with Tukey's multiple comparison test. For vacuole analysis, one-way ANOVA with Tukey's multiple comparison test (Figs 3 and 4) or unpaired Student's *t*-test with Welch's correction, two-tailed (Fig. 5) was used to determine significance. All statistical analyses were performed using Prism, version 6.0 (GraphPad Software v8.4). All experiments were performed in biological triplicates. Significance is defined as *P*<0.05, and bars in graphs represent the mean±s.d.

## Supplementary Material

10.1242/dmm.049693_sup1 Supplementary information
