# Supplementary material for: String/Cdc25 phosphatase is a suppressor of Tau-associated neurodegeneration
Source: Dis Model Mech. 2023 Jan 23;16(1):dmm049693. doi: 10.1242/dmm.049693 (PMC9903143; doi:10.1242/dmm.049693)
Supplement: Supplementary information [file dmm-16-049693-s1.pdf]

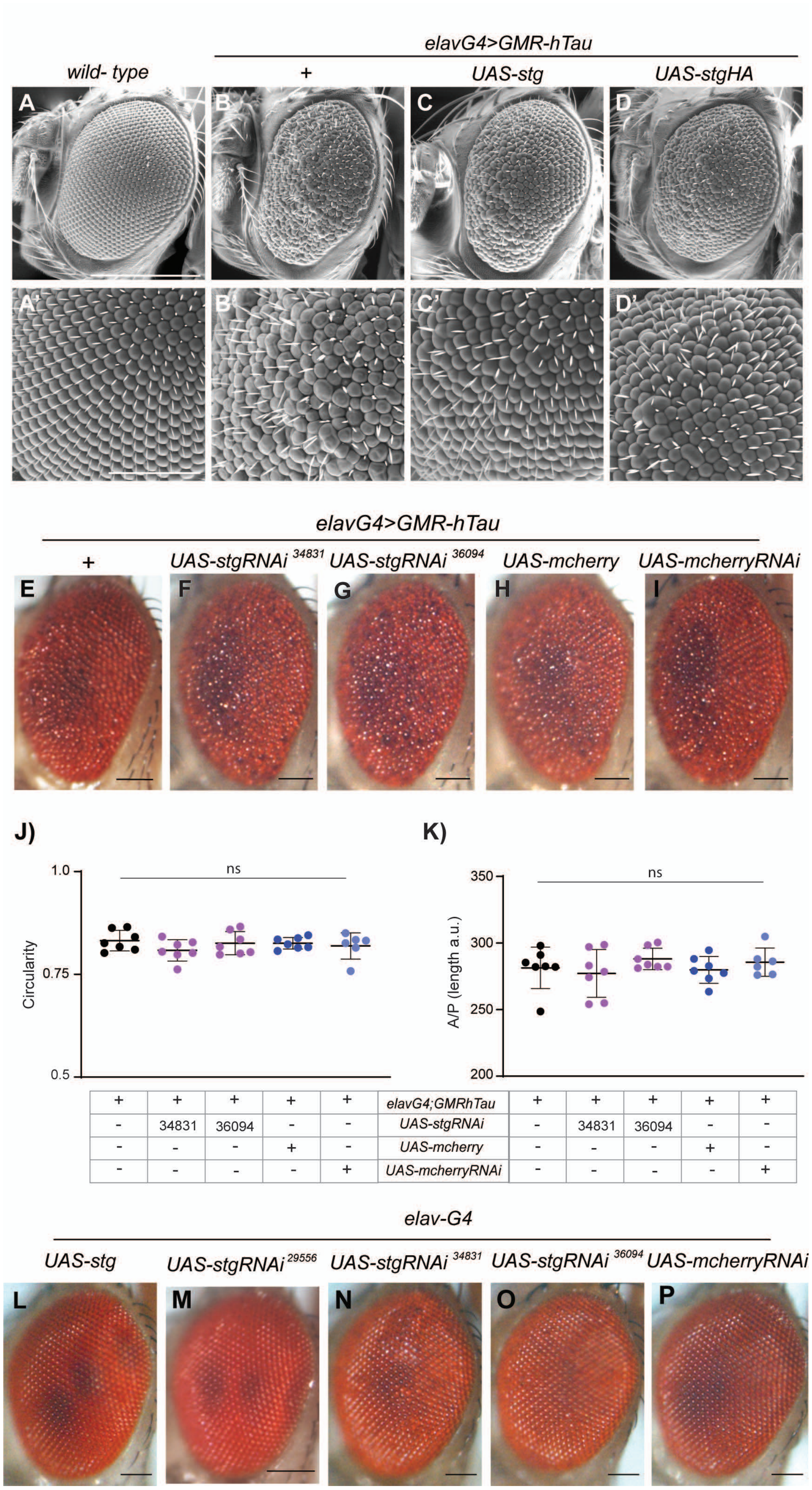


Disease Models & Mechanisms • Supplementary information

**Fig. S1. Stg suppresses the rough eye phenotype of hTau expression flies** (A – D) Representative scanning electron microscopy images of the retina of (A) control flies, (B) flies expressing one copy of hTau (*elavG4>GMR-hTau;* +), and (C,D) flies co-expressing Stg and hTau ( *elavG4>GMR-hTau; stg, and elavG4>GMR-hTau; stg-HA)*. Scale bar is 300 µm. (A'- D') magnified views of A, B, C and D, respectively. Scale bar is 100 µm(E-I) Representative images of the retina from (E) flies expressing one copy of hTau (*elavG4> GMR-hTau;* n=7), (F, G) GMR-hTau flies co-expressing UAS-StgRNAi (*elavG4> GMR- hTau; StgRNAi*; n=7) and elavG4> GMR-hTau flies co- expressing UAS control lines (H) UAS-mCherry (n=7) and (I) UAS-mCherryRNAi (n=6). Scale bar is 100 µm (J, K) Quantification of the circularity (J) and anterior -posterior axis length (K) in the indicated genotypes. Image quantification was performed on Fiji-ImageJ and the results were analyzed using one-way ANOVA, multiple comparisons. (L-P) Representative images of the retina of flies expressing UAS-Stg (*elavG4> UAS-stg*) (L) and UAS-stgRNAi alleles (elavG4> UAS-stgRNAi) (M-O) under the control of elavG4. UAS-mcherryRNAi (P) was used as control. Scale bar is 100 µm.

Disease Models & Mechanisms • Supplementary information

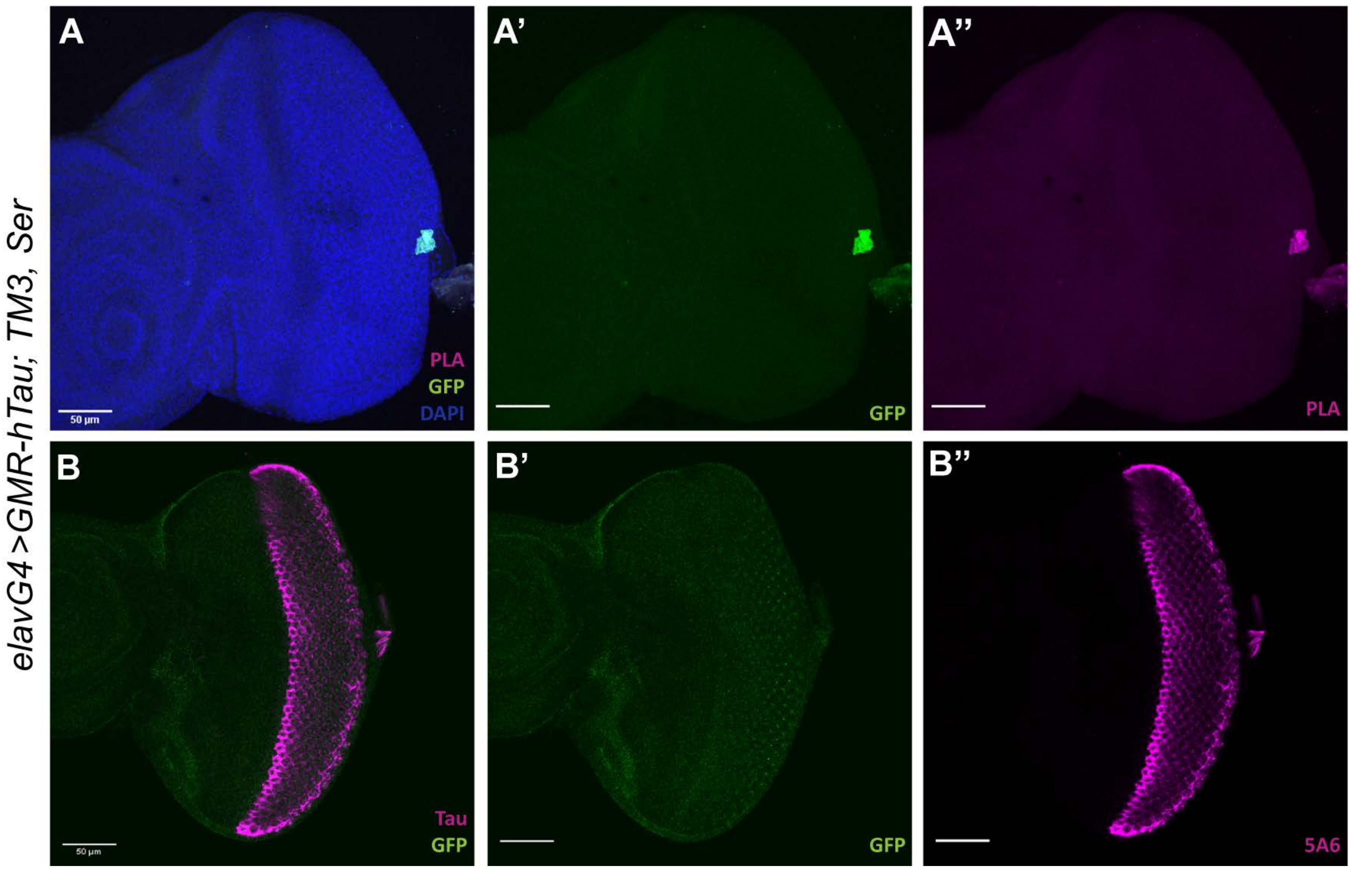


**Fig. S2. Negative control for PLA experiment**. Representative images of eye imaginal discs from *elavG4> GMRhTau;TM3,Ser* larvae stained for Tau (B, magenta) and showing absence of endogenous stg-GFP.

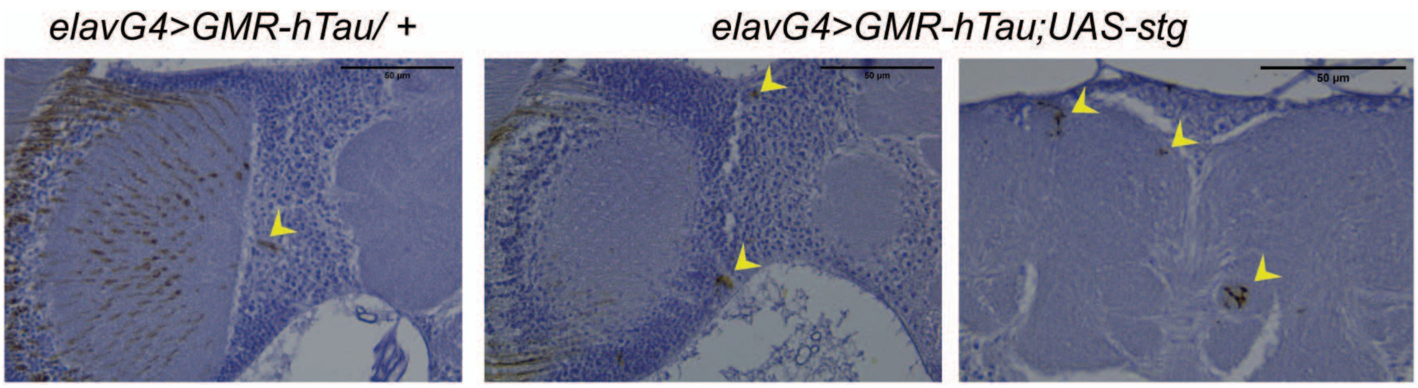


**Fig. S3.** Analysis of phosphorylated Tau (Ser202/Thr205; AT8 antibody) in cross-sections of adult heads for the indicated genotypes. Yellow arrowheads indicate Tau deposition outside the domain of GMR-hTau expression. Scale bar is 50 µm.

Disease Models & Mechanisms • Supplementary information

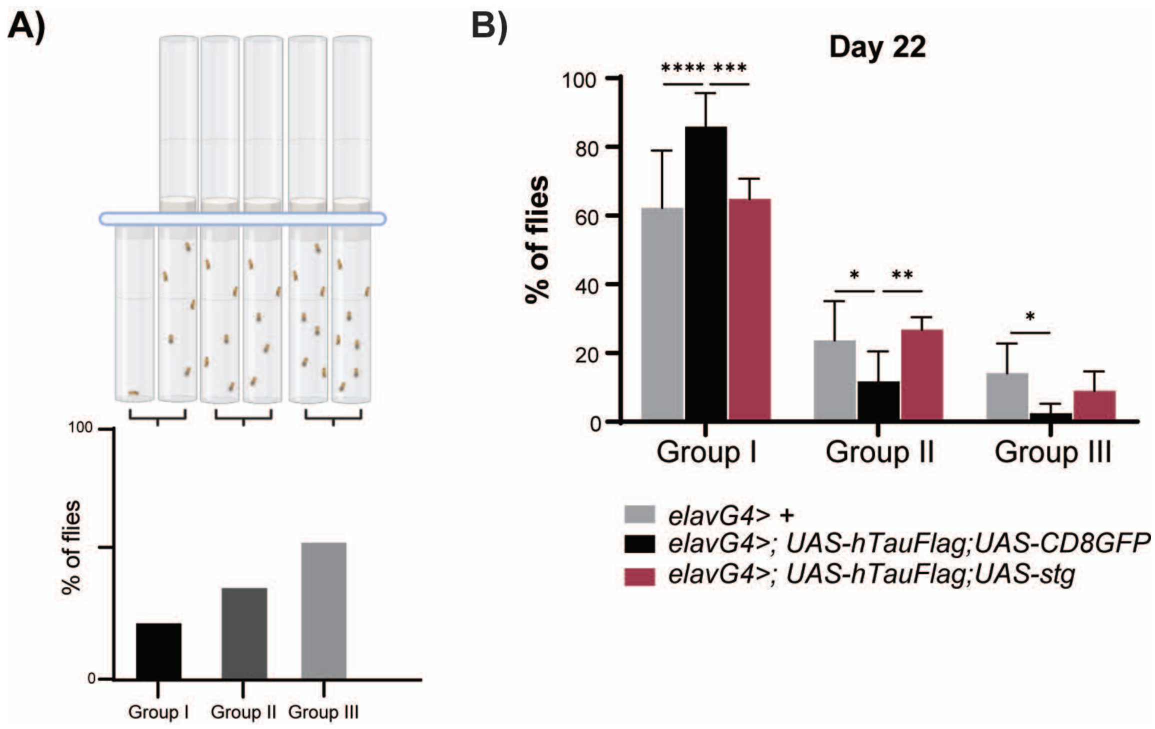


**Fig. S4. Graphical representation of the climbing assay with countercurrent apparatus.**

(A) Final distribution of the flies in the countercurrent apparatus represented as a bar graph. (B) Quantification of the percentage of flies in each group for *elavG4> GMR-hTau;TubGal80*$^{ts}$ (n= 246) and *elavG4; GMR-hTau,UAS-Stg;TubGal80*$^{ts}$ (n= 191), at day 22. Statistical significance was calculated using two-way ANOVA, multiple comparisons. Error bars denote SD; * $p < 0.05$; ** $p < 0.01$; *** $p < 0.0001$.

Disease Models & Mechanisms • Supplementary information

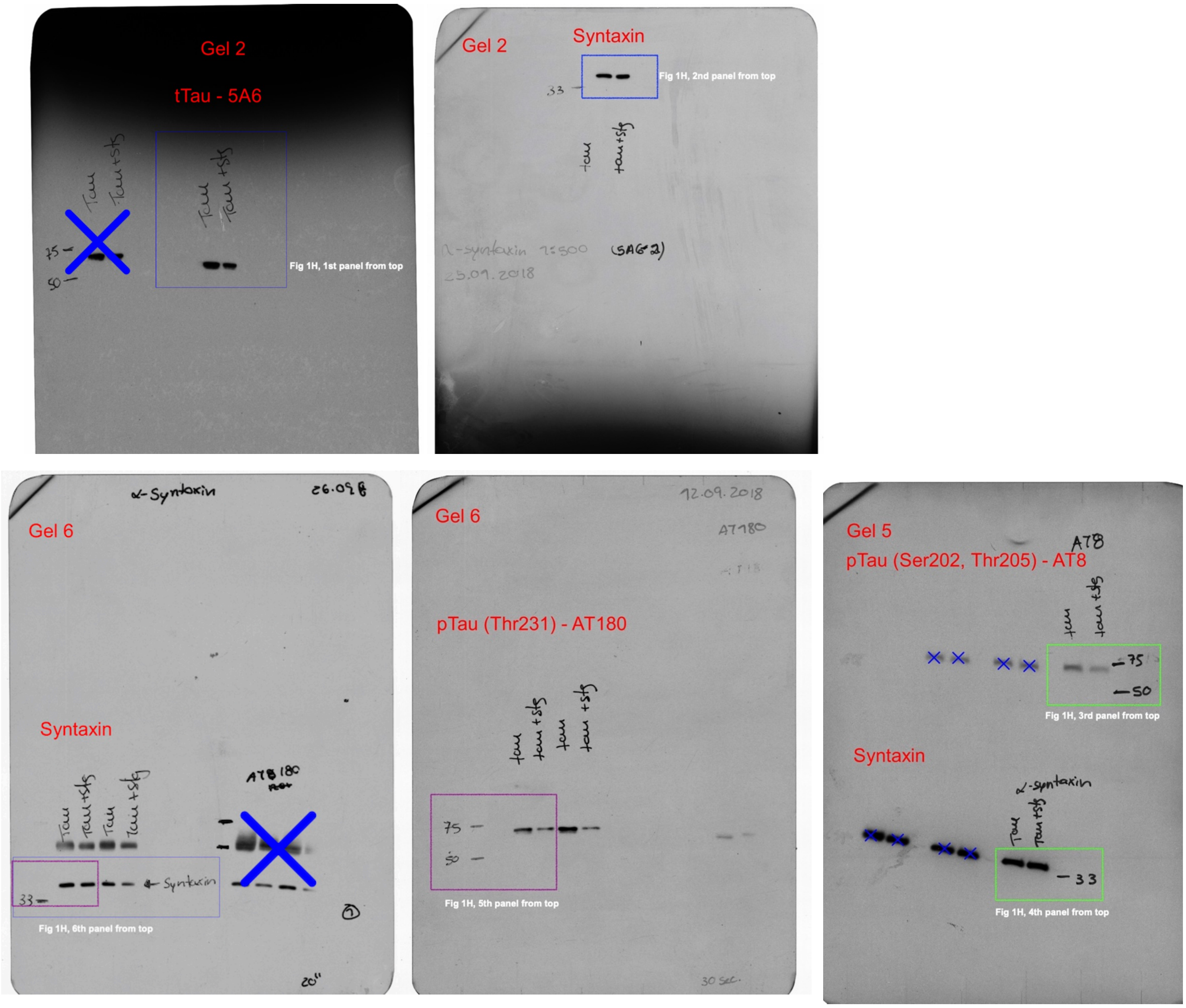


**Fig. S5. Raw data of western blots used in Figure 1H.**

Disease Models & Mechanisms • Supplementary information

**Table S1.** ***Drosophila*** **genotypes used in this study**

| STOCK | GENOTYPE | SOURCE |
|---|---|---|
| **GMR-Gal 4** | | In house |
| ***elav*-Gal 4** | P{w[+mW.hs]=GawB}*elav*$^{[C115]}$;; | BDSC (#458) |
| ***elav*-Gal 4; GMR::hTau** | P{w[+mW.hs]=GawB}*elav*$^{[C155]}$; P{w[+mC]=GMR-htau.Ex}1.1 | BDSC (#51360) |
| **UAS-*string*** | w[1118]; P{w[+mC]=UAS-*stg*.N}16/CyO,P{ry[+t7.2]=sevRas1.V12}FK1 | BDSC (#4777) |
| **UAS- *string* $^{RNAi}$** | y[1] v[1]; P{y[+t7.7] v[+t1.8]=TRiP.JF03235}attP2 | BDSC (#29556) |
| **UAS- *string* $^{RNAi}$** | y[1] sc[*] v[1] sev[21]; P{y[+t7.7] v[+t1.8]=TRiP.GL00513}attP40 | BDSC (#36094) |
| **UAS- *string* $^{RNAi}$** | y[1] sc[*] v[1]; P{y[+t7.7] v[+t1.8]=TRiP.HMS00146}attP2 | BDSC (#34831) |
| **UAS- *string* phosphatase-dead** | W; Sp/Cyo ; P{w[+mC]=UAS-*stg*$^{C379S}$/TM6B | In house |
| **OR-R** | Oregon-R | In house |
| **UAS-*stg*/CyO; TubGal80$^{ts}$/TM6B** | W; UAS-*stg*/CyO; *tub*PGal80$^{ts}$/TM6B | In house |
| **TubGal80$^{ts}$** | w[*]; P{w[+mC]=*tub*P-GAL80[ts]}2/TM2 | BDSC (#7017) |
| **Stg-GFP/TM3** | y[1] w[*]; PBac{y[+mDint2]=HpaI-GFP.A}*stg*$^{[YD0685]}$/TM6C, Sb[1 | BDSC (#50879) |
| **UAS-m-CD8GFP** | y[1] w[*]; betaTub60D[Pin-Yt]/CyO; P{w[+mC]=UAS-mCD8::GFP.L}LL6 | BDSC (#5130) |
| **UAS-mCherry** | y[1] sc[*] v[1] sev[21]; P{y[+t7.7] v[+t1.8]=UAS-mCherry.VALIUM10}attP2 | BDSC (#35787) |
| **UAS-mCherryRNAi** | y[1] sc[*] v[1] sev[21]; P{y[+t7.7] v[+t1.8]=VALIUM20-mCherry}attP2 | BDSC (#35785) |

Disease Models & Mechanisms • Supplementary information
